# A non-injected opioid analgesia protocol for acute pain crisis in adolescents and adults with sickle cell disease

**DOI:** 10.1177/20494637211033814

**Published:** 2021-08-02

**Authors:** Paul Telfer, Jonathan Bestwick, James Elander, Arlene Osias, Nosheen Khalid, Imogen Skene, Ruben Nzouakou, Joanne Challands, Filipa Barroso, Banu Kaya

**Affiliations:** 1 Centre for Genomics and Child Health, Blizard Institute, Queen Mary University of London, London, UK; 2 Department of Hematology, Royal London Hospital, Barts Health NHS Trust, London UK; 3 Wolfson Institute of Preventive Medicine, Queen Mary University of London, London, UK; 4 School of Psychology, University of Derby, Derby, UK; 5 Childrens Research Facility, Royal London Hospital, Barts Health NHS Trust, London, UK; 6 Emergency Medicine Research Facility, Royal London Hospital, Barts Health NHS Trust, London, UK; 7 Department of Anesthetics, Royal London Hospital, Barts Health NHS Trust, London, UK

**Keywords:** Sickle, crisis, pain, opioid, VOC, analgesia

## Abstract

Initial management of the acute pain crisis (APC) of sickle cell disease (SCD) is often unsatisfactory, and might be improved by developing a standardised analgesia protocol. Here, we report the first stages in developing a standard oral protocol for adolescents and adults. Initially, we performed a dose finding study to determine the maximal tolerated dose of sublingual fentanyl (MTD SLF) given on arrival in the acute care facility, when combined with repeated doses of oral oxycodone. We used a dose escalation algorithm with two dosing ranges based on patient’s weight (<50 kg or >50 kg). We also made a preliminary evaluation of the safety and efficacy of the protocol. The study took place in a large tertiary centre in London, UK. Ninety patients in the age range 14–60 years were pre-consented and 31 treatment episodes were evaluated. The first 21 episodes constituted the dose escalation study, establishing the MTD SLF at 600 mcg (>50 kg) or 400 mcg (<50 kg). Further evaluation of the protocol indicated no evidence of severe opioid toxicity, nor increased incidence of acute chest syndrome (ACS). Between 0 and 6 hours, the overall gradient of reduction of visual analogue pain score (visual analogue scale (VAS)) was 0.32 centimetres (cm) per hour (95% confidence interval (CI) = 0.20 to 0.44, p < 0.001). For episodes on MTD SLF, there was median (interquartile range (IQR)) reduction in VAS score of 2.8 cm (0–4.2) and 59% had at least a 2.6-cm reduction. These results are supportive of further evaluation of this protocol for acute analgesia of APC in a hospital setting and potentially for supervised home management.

## Introduction

The acute pain crisis (APC) is the commonest acute complication of sickle cell disease (SCD).^1^ Severe episodes are distressing, disruptive to normal activity, associated with life-threatening complications such as acute chest syndrome (ACS), and may predispose to chronic pain.^2^ Initial management in the emergency department (ED) or other acute care setting requires rapid assessment and administration of first dose of analgesia, usually opioid, within 30–60 minutes.^3–5^ Afterwards, repeated doses of analgesia are generally required, together with mandatory monitoring in order to ensure adequacy of analgesia, to anticipate additional complications of APC and to avoid opioid toxicity.^3,5,6^ There is no standard analgesia protocol for paediatric, adolescent or adult patients and controversy remains around a number of aspects of the treatment pathway including choice of opioid, timing and route of administration, and differences in practice between children, adolescents and adults. This prompted the National Heart Lung and Blood Institute (NHLBI) panel in the United States^3,7^ and the National Institute of Clinical and Health Care Excellence (NICE) in the United Kingdom^4^ to recommend studies to determine optimal management of APC.

Morphine, the most commonly used opioid,^8^ is a mu receptor agonist, metabolised mainly through glucuronidation by the enzyme UGT2B7. The resulting metabolites include morphine-6-glucuronide (M6G), which is pharmacologically active and has been associated with an increased risk of ACS.^9^ Oxycodone and fentanyl are strong opioids which differ from morphine in pharmacokinetics, undergoing extensive first-phase metabolism via CYP2D6 and CYP3A4 pathways, enzymes which are potentially activated or inhibited by a range of drugs that could affect the therapeutic response.^10^ Oral oxycodone is commonly used for home management of pain in the United States,^11^ but might be a suitable alternative to morphine for management of acute sickle cell pain in patients who have not been heavily exposed to strong opioids.

With regard to the route of administration, the intravenous route is generally accepted as the gold standard, but there are a number of considerations which justify efforts to develop non-intravenous opioid protocols in both paediatric and adult SCD populations. First, delays in administration of analgesia often occur because of poor venous access. Second, there is evidence from the addiction literature both from animal studies and human observation studies, that rapid and frequent elevations in plasma and brain drug levels during intravenous administration are more likely to induce tolerance and dependency.^12^ Although evidence for this effect in patients with SCD is lacking, we believe that there is cause for concern about adverse effects associated with repeated doses of intravenous opioids given over a prolonged period in this patient group. Studies in children which show that a standardised oral-based opioid protocol can be effective and safe^13,14^ suggest that this kind of protocol could also be evaluated in adolescents and adults.

Opioid drugs can be rapidly absorbed through the oral and nasal mucosa, leading to a rapid rise in plasma drug levels.^15^ For instance, the onset of action for sublingual fentanyl is 8–10 minutes and peak effect at 40–60 minutes.^16^ The transmucosal route of administration could potentially enable rapid action of the first dose of opioid analgesia, avoiding delays entailed with intravenous administration. By restricting transmucosal opioid to the first dose only, the potential risks associated with sharp elevations in plasma and brain opioid levels may be mitigated. Intranasal and buccal fentanyl have been used to manage acute sickle pain in children and adults, but it is not clear how best to incorporate additional rapid-acting opioid analgesia into a standard protocol for acute pain.^6,17,18^ We have previously shown that intranasal diamorphine (IND) given as a single acute dose in combination with a pre-scheduled protocol of oral morphine can provide effective analgesia in children and potentially reduce the time to first analgesia compared to intravenous opioid.^14,19^ Some problems with use of IND were identified. First, 35% of children found IND uncomfortable, particularly adolescents, who are administered a higher concentration of diamorphine solution because of their greater weight. Second, diamorphine is not available in some health care systems and uptake might be limited by perceived association with drug dependency.

The overarching aim of our programme of work is to develop a standard oral-based analgesia protocol, suitable for rapid administration in the ED. In this study, we assessed the maximal safe dose of sublingual fentanyl (Abstral^®^) given on arrival in the acute health care facility, when combined with a programmed dosing schedule of oral oxycodone. Oxycodone was chosen in this protocol building on our previous experience of using oxycodone as a replacement opioid in adolescent and adult patients treated in our institution who had adverse effects, or poor pain control with morphine. The study also aimed to obtain preliminary data on efficacy and safety which could be used to design a follow-up randomised controlled trial comparing this protocol with standard analgesia.

## Methods

### Protocol development

**Figure 1.**
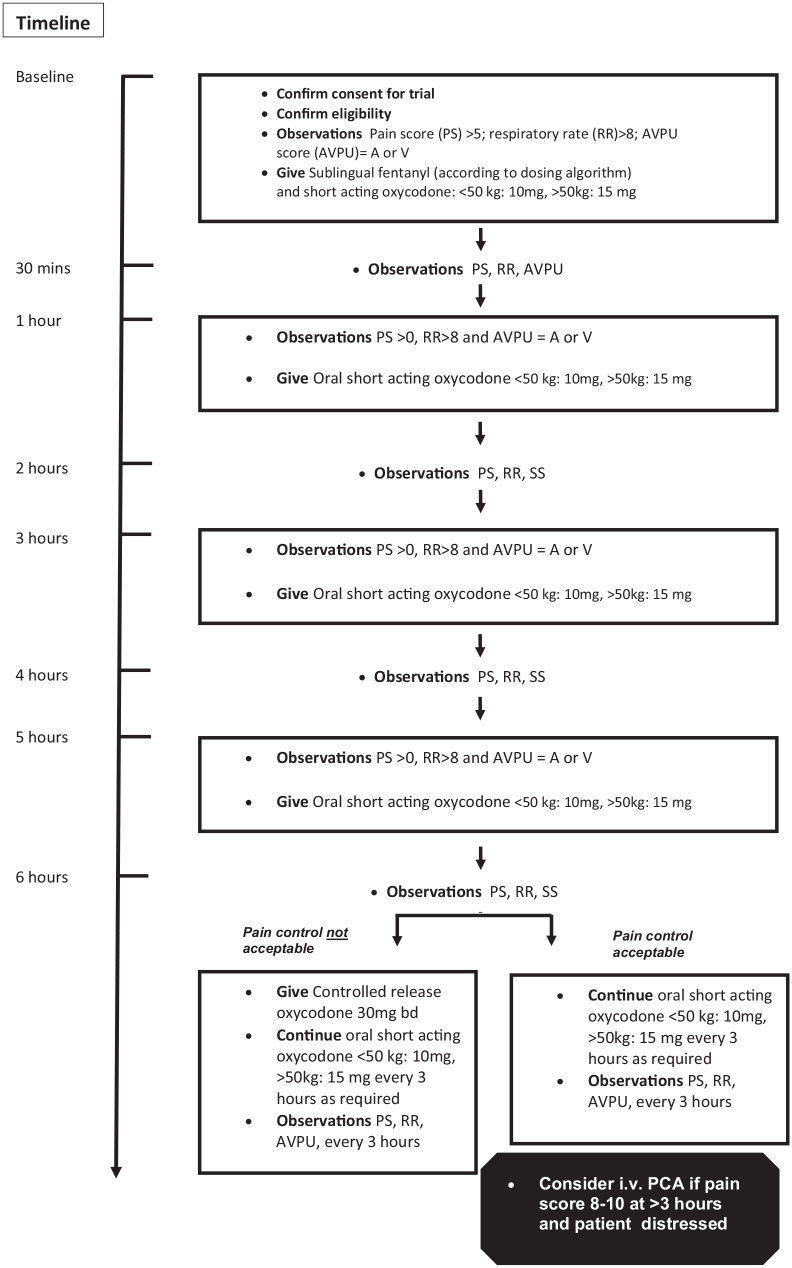
Treatment and observation flow chart. PS: pain score, RR: respiratory rate, A: alert, V: responds to verbal commands, P: responds to painful stimuli, U: unresponsive, PCA: patient-controlled analgesia.

Our standard institutional analgesia protocols are based on short-acting oral morphine for breakthrough and controlled-release morphine for background analgesia. Some adults, particularly those with more severe or frequent hospitalisations, are treated with subcutaneous injections combined with controlled-release oral long-acting opioid for background analgesia. The study was part of a long-term programme of work in our service to improve pain management and patient experience, involving consultation with patients, families and the wider SCD multi-disciplinary team.^14,19–21^ We had previously developed a protocol for children to ensure rapid onset of analgesia and sustained analgesia without using injected opioids. We used a single dose of transmucosal opioid (IND) on first arrival in ED, to obtain rapid increase in plasma drug concentration and rapid analgesic effect. The pharmacokinetics of oral short-acting opioid are slower than for transmucosal, but we predicted that if the oral short-acting opioid is administered simultaneously with transmucosal, drug levels should reach analgesic levels simultaneously with the decrease in drug levels of transmucosal opioid.^15^ In this way, we aimed to ensure sustained opioid drug levels and analgesia effect during the first few hours of pain management. In this study, we modified this analgesia protocol for use in adolescents and adults by replacing IND with sublingual fentanyl (SLF; Abstral^®^, Kyowa Kirin). For breakthrough analgesia, short-acting oral morphine was replaced with oral short-acting oxycodone (OxyNorm^®^, Mundipharma), and for background analgesia, controlled-release morphine was replaced with controlled-release oxycodone (OxyContin^®^, Mundipharma). The protocol is shown in Figure 1.

### Consent, inclusion and exclusion criteria

Patients were consented in the out-patient setting and consent confirmed on presentation with APC. Inclusion and exclusion criteria are shown in Table 1. We excluded those weighing less than 35 kg and those who were taking strong opioids (as defined in the NICE guideline)^4^ as a regular daily analgesic prescription at home. There was no limit to the annual rate of admissions with APC prior to participation in the study.

**Table 1.** Inclusion and exclusion criteria.

*Inclusion criteria*
At consent
1. Diagnosis of SCD (any genotype)
2. Aged 14–60 years
At confirmation of consent
Points 1–2 as above plus
1. VOC requiring hospital treatment
2. Pain score 5 or more on verbal 1–10 scale
3. Part 1: no more than one previous pain crisis treated with trial protocol; Part 2: no more than two previous pain crises treated with trial protocol
*Exclusion criteria*
At consent
1. Weight <35 kg
2. History of allergic reaction to fentanyl or oxycodone or their excipients
3. Severe hepatic or renal impairment
4. Regular daily home medication with strong opioids
5. Administration of CYP3A4 inhibitor
6. Concurrent administration of Selective Serotonin Re-uptake Inhibitor (SSRI) or a Serotonin Norepinephrine Re-uptake Inhibitor (SNRI) or monoamine oxidase inhibitor (MAOI) within previous 2 weeks
7. Documented history of clinically significant brain tumour
8. History of severe symptomatic chronic obstructive airways disease or chronic asthma
9. History of pulmonary hypertension
10. Chronic constipation
11. Pregnant or breastfeeding
12. Unable to understand spoken or written English
At time of confirmation of consent
Points 1–12 as above plus:
1. Administration of strong opioid after arrival in acute care facility (ED or HDU) prior to enrolment in SCAPE protocol
2. Respiratory rate >28/min or <8/min
3. AVPU score = P or U
4. Blood pressure <80 systolic
5. Pulse rate <50/min
6. Uncontrollable vomiting
7. Hypovolaemia
8. Acute abdominal complication requiring surgical intervention
9. Paralytic ileus
10. Delayed gastric emptying
11. Clinical suspicion of stroke
12. Documented history of head injury
13. Raised intracranial pressure
14. Fulminant priapism in men
15. Ingestion of excessive alcohol within 12 hours of study entry
16. Ingestion of CNS depressant other than medication to treat VOC within 12 hours of study entry

SCD: sickle cell disease; HDU: haematology day unit; ED: emergency department; AVPU: Alert, responds to Verbal instructions, Painful stimuli or Unresponsive; CNS: central nervous system.

### Study procedures

In the event of APC requiring treatment in hospital, the patient or parent/guardian was asked to phone the trial centre to inform of their intention of attending. Children were directed to the paediatric ED, adults (age >16 years) either to the adult ED or, if there was a bed available, to the haematology day unit (HDU). The trial was implemented with the assistance of a dedicated nurse and a trial manager. Paediatric and emergency medicine clinical research teams contributed to delivery of the trial in their respective areas. Monitoring and treatment over the first 6 hours were done by the trial nurse, while management after 6 hours was undertaken by standard care clinical and nursing staff. The intention was to continue oral analgesia according to the trial protocol, after the first 6 hours of treatment on the protocol, but there was an option to switch to alternative oral opioid or injected opioid using institutional protocols. Ibuprofen and paracetamol at standard recommended dosage were routinely prescribed and administered as additional analgesic medication.

### Part 1: determination of MTD SLF

Sequential dosing of groups of three episodes was undertaken using a ‘group up-and-down’ algorithm,^22^ with two dose options depending on the patient’s weight (<50 kg or >50 kg). The dose of short-acting oxycodone was fixed at 10 mg (<50 kg) and 15 mg (>50 kg). The range of doses of fentanyl is shown in Table 2. For safety monitoring, we used indicators adapted from the National Early Warning Score (NEWS, https://www.rcplondon.ac.uk/projects/outputs/national-early-warning-score-news-2). These consisted of respiratory rate (RR) and sedation according to the AVPU scale (Alert, responds to Verbal instructions, Painful stimuli or Unresponsive). The lowest anticipated effective dose was initially used and after three episodes had been completed, the trial safety monitoring committee (TSMC) assessed safety at time points t = 0, 30 minutes, 1 hour and hourly up to 6 hours. A patient was classified as intolerant if the AVPU score was P or U, or if RR was below 8 per minute at any of these time points. If all patients were tolerant, the dose was increased by one iteration. If one or more patients were intolerant, the dose was reduced by one increment. The MTD was to be the most commonly used dose after 21 adjudicated episodes.

**Table 2.** Dosage algorithm for maximal tolerated dose (MTD) of sublingual fentanyl.

Patient weight	Dosage iteration				
	−1	Starting	+1	+2	+3
30–50 kg	100 mcg	100 mcg	200 mcg	300 mcg	400 mcg
>50 kg	100 mcg	200 mcg	300 mcg	400 mcg	600 mcg

### Part 2: further data collection using MTD SLF

After completion of the dose finding study, we aimed to obtain further data on safety and efficacy using MTD SLF to determine suitable end points for a controlled trial. Although not formally powered, the protocol envisaged a total of 30 episodes at MTD (including episodes treated during both Parts 1 and 2), but the study was actually terminated after a total of 22 episodes at MTD SLF, due to funding constraints.

### Patient-reported outcomes

Patient satisfaction was elicited by verbal and written feedback as part of the protocol-specified follow-up 28 days after discharge.

### Safety monitoring and adverse event reporting

The main safety parameters were RR and sedation score. These were evaluated systematically for the first 6 hours after administration of study medication. After 6 hours, safety parameters were evaluated 3-hourly by the care team according to standard institutional protocols and these were recorded on standard institutional observations charts, reviewed by the trial team over time period 6–24 hours, and daily thereafter until discharge from hospital. Other opioid adverse effects (nausea and vomiting, pruritis, constipation and urinary retention) were monitored at baseline, 3 and 6 hours and then averaged daily until discharge from hospital, and graded on a 0–4 scale based on a published terminology of categories (https://ctep.cancer.gov/protocolDevelopment/electronic_applications/docs/ctcaev3.pdf).

Definitions of severe adverse events were specified in the protocol to take account of the acute nature of APC and likely admission to hospital. These included abnormally prolonged hospital stay (more than 14 days) and potentially life-threatening complication of SCD including acute stroke, acute complication requiring exchange transfusion or admission to intensive care unit.

### Funding and trial authorisations

The trial was funded by a grant from the Barts Charity (reference no. 1704), the National Institute for Health Research North Thames Clinical Research Network Divisional Contingency Funding, and unrestricted grants from Kyowa Kirin and Napp Pharmaceuticals. It was registered under the European Directory of Clinical Trials with reference number 2013-004161-14, and sponsored by Barts Health NHS Trust and Queen Mary University of London. Approvals for the initial protocol and subsequent amendments were obtained from the London City and East Research Ethics Committee (reference no. 14/LO/0165), the UK Medicines and Health Products Regulatory Agency (protocol no. 008414) and the National Health Research Authority. Details of protocol amendments during the study are given in Supplementary Appendix 1. The study was adopted by the National Institute for Health Research (NIHR) Clinical Research Network trials portfolio and conducted with the assistance of Pediatric and Emergency Medicines research teams at our institution.

### Statistical analysis

MTD of SLF administered was considered the primary end point. A number of protocol-defined efficacy and safety end points were evaluated for potential use in a subsequent controlled trial. These were principally focussed on the first 6 hours of treatment, but data were also collected beyond 6 hours for analysis of overall efficacy and safety during admission and after discharge. Visual analogue scale (VAS) pain score between baseline and 6 hours was analysed using time series regression analysis. Predictor factors were MTD versus not MTD, sex, paediatric versus adult and weight >50 kg or <50 kg.

## Results

### Participants

**Figure 2.**
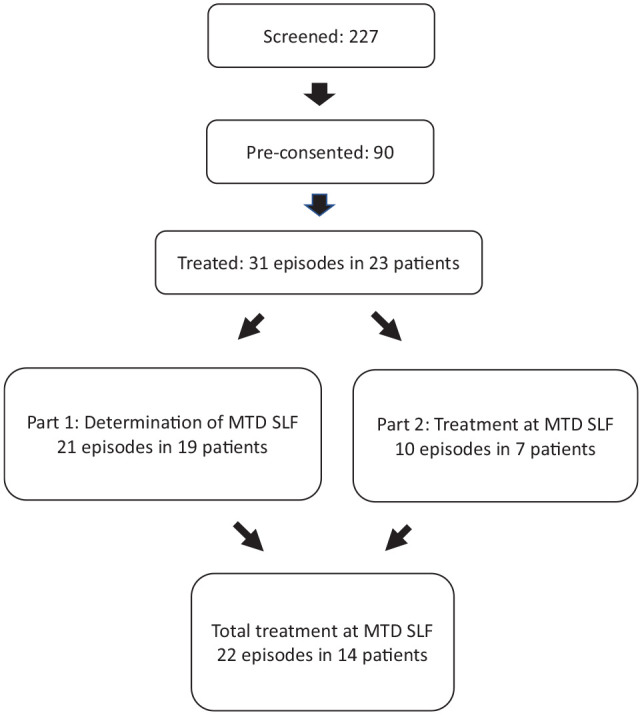
Consort study diagram.

Ninety patients were consented. Overall, 31 treatment episodes in 23 patients were evaluated, including 21 episodes in 19 patients in Part 1 for determination of MDT SLF and a further 10 episodes in seven patients at MTD SLF in Part 2. Sixty-three episodes in 34 consented patients were not evaluated. Forty (63.5%) of these episodes were treated outside of normal working hours of the trial team and 23 (36.5%) did not meet inclusion criteria. Reasons for not meeting inclusion criteria included presentation for causes unrelated to pain, administration of alternative opioid analgesia in ED, taking excluded additional medication and previously treated more than permitted number of times in study. Excluded episodes were treated with standard institutional analgesia protocols. Altogether, including episodes in Parts 1 and 2, 22 treatment episodes in 14 patients were evaluated at MTD SLF. The consort diagram for the study is shown in Figure 2, the baseline clinical features of study participants are shown in Table 3, and the clinical features of the episodes are shown in Table 4. Seven (30%) were under 18 years of age, four (17%) were under 50 kg in weight and as per protocol received the lower dose of SLF and oxycodone. Prior to arrival in hospital, opioids were administered for 22/31 (71%) of episodes. This includes moderate and strong opioids taken at home and opioid administered in the ambulance in those who required ambulance transport. Ten episodes were initially treated in our HDU and 21 in ED.

**Table 3.** Clinical features of study patients.

	Sub-MTD SLF 9 patients	MTD SLF 14 patients	Total 23 patients
Demographics			
Age, years, median (range)	19 (16–38)	21 (12–41)	21 (12–41)
Age <18 years, number (%)	3 (33)	4 (29)	7 (30)
Female, number (%)	3 (33)	7 (50)	10 (44)
Weight <50 kg, number (%)	1 (11)	3 (21)	4 (17)
Genotype HbSS, number (%)	9 (100)	13 (93)^a^	22 (96)^a^
Treatment			
Hydroxycarbamide, number (%)	2 (22)	7 (50)	9 (39)
Regular transfusion, number (%)	2 (22)	2 (14)	4 (17)
Acute pain history			
Annual admissions, median (range)^b^	3 (0–14)	4 (0–16)	4 (0–16)
Annual home-managed, median (range)^c^	32 (4–96)	7 (0–30)	24 (0–120)
Home opioid usage^d^			
Moderate strength opioid^e^			
Number (%)	7 (78)	10 (71)	17 (71)
Median days per month (range)	8 (0–14)	1 (0–14)	1 (0–14)
Strong opioid^e^			
Number (%)	4 (44)	5 (36)	9 (38)
Median days per month (range)	0 (0–7)	0 (0–10)	0 (0–10)
Usual hospital analgesia protocol, number (%)			
Intranasal diamorphine, oral short-acting morphine and controlled-release morphine	0 (0)	1 (7)	1 (4.3)
Fentanyl lozenge, oral short-acting morphine and controlled-release morphine	7 (78)	8 (57)	15 (65)
Subcutaneous morphine and controlled-release morphine	2 (22)	3 (21)	5 (21)
Subcutaneous oxycodone and controlled-release oxycodone	0 (0)	1 (7)	1 (4)

MTD SLF: maximal tolerated dose of sublingual fentanyl.

a One patient was HbSC.

b Averaged over 24 months prior to consent.

c Calculated from self-report over previous 3 months at consent.

d Self-report over previous month, at consent.

e Moderate strength opioids include codeine, dihydrocodeine and tramadol, and strong opioids include morphine formulations and oxycodone.

**Table 4.** Clinical features of treated acute pain crises.

	Sub-MTD SLF (n = 9)	MTD SLF (n = 22)	Total (n = 31)
Site of pain			
Extremities^a^	6 (67)	16 (73)	22 (71)
Back	4 (44)	13 (59)	17 (55)
Chest	3 (33)	7 (32)	10 (33)
Head	1 (11)	3 (14)	4 (13)
Abdo	0 (0)	5 (23)	5 (16)
Analgesia taken at home prior to attending hospital			
Paracetamol	5 (56)	15 (68)	20 (65)
NSAID	5 (56)	9 (41)	14 (45)
Moderate strength opioid^b^	3 (33)	14 (67)	17 (55)
Strong opioid^c^	3 (33)	3 (14)	6 (19)
Mode of transportation to hospital			
Ambulance	5 (56)	3 (14)	8 (25.8)
Car	2 (22)	8 (36)	10 (32)
Public transport	2 (22)	11 (50)	13 (423)
Physical signs at presentation			
Jaundice	8 (89)	21 (96)	29 (94)
Pallor	2 (22)	18 (82)	20 (65)
Respiratory signs	0 (0)	2 (9)	2 (6)
Abdominal signs	0 (0)	3 (14)	3 (10)
Priapism	0 (0)	0 (0)	0 (0)
Neurological signs	0 (0)	1 (5)	1 (3)
Other^d^	1 (11)	3 (14)	4 (13)
Vital signs at presentation			
	Mean (range)	Mean (range)	Mean (range)
Verbal pain score (1–10)	7 (6–9)	8 (5–10)	7 (5–10)
Respiratory rate (per min)	17 (12–19)	20 (14–28)	19 (12–28)
Sedation (AVPU) score^e^	A in all cases	A in all cases	A in all cases
Pulse rate (per minute)	82 (60–111)	86 (67–129)	85 (60–129)
Oxygen saturation (%)	96 (87–100)	96 (92–100)	96 (87–100)
Temperature (°C)	36.4 (35.1–37.6)	37 (36.1–38.8)	37 (35.1–38.8)
Blood pressure (systolic)	116 (97–140)	124 (94–153)	122 (94–153)
Blood pressure (diastolic)	63 (52–77)	68 (49–93)	66 (49–93)

MTD SLF: maximal tolerated dose sublingual fentanyl; NSAID: non-steroidal anti-inflammatory.

Figures in parentheses are the percentages.

a Extremities includes L or R arm, L or R leg.

b Moderate strength opioid: codeine phosphate, dihydrocodeine and tramadol.

c Strong opioid: oral short-acting or controlled-release morphine and oral short-acting or controlled-release oxycodone.

d Tender areas on extremities in four cases.

e A: alert; V: responds to verbal commands; P: responds to painful stimuli; U: unresponsive.

### Determination of maximal tolerated dose of SLF

SLF dose was increased sequentially up to the maximum specified on the algorithm (600 mcg for >50 kg or 400 mcg for <50 kg). This being the most frequently used dose, it met the criteria for MTD SLF. During the first 6 hours of treatment, there was no evidence of respiratory depression (RR <8/min) and no significant difference in RR at different dose increments. There was one episode associated with sedation (AVPU score of P in episode 11), but this episode was a presentation with severe ACS; sedation occurred after switching to intravenous opioid analgesia and was associated with hypoxaemia due to acute pulmonary sickling. This was reversed with urgent exchange transfusion instituted within 6 hours of triage.

### Dosing of short-acting oxycodone

The analgesia protocol included pre-specified doses of short-acting oxycodone during the first 6 hours. All patients received the initial oxycodone dose simultaneously with SLF, but at subsequent time points, scheduled doses were not given in some cases. The main reasons for withholding doses were physician decision that the patient was becoming too drowsy (AVPU score of ‘V’, responds to verbal commands), and particularly towards the end of the 6-hour study period because pain had settled and the patient was considered ready for discharge.

### Efficacy

Median verbal pain score on presentation was 7 (range 5–10) (Table 3). After confirmation of consent and trial enrolment, pain score was re-evaluated by VAS prior to first treatment (t = 0). Median VAS score was 6.5 cm (range = 2.7–10). This difference in pain reported verbally compared to VAS may relate to variations in patient behaviour in reporting verbally compared to VAS, or to delayed effects of analgesia administered at home or in transit, prior to treatment in the study.

Thereafter, the gradient of reduction of VAS pain score was 0.32 cm per hour (95% confidence interval (CI) = 0.20 to 0.44, p < 0.001). There was no significant difference in gradient between patients on MDT SLF and sub-MDT SLF, between males and females, and between those in the highest and lowest quartiles for age and weight. Further efficacy end points are detailed in Table 5. For MTD SLF, median reduction (interquartile range (IQR)) in VAS score from t = 0 to 6 hours was 2.8 cm (0–4.15) and 59% had a reduction of at least 2.6 cm. Three episodes in two patients required intravenous opioid via patient-controlled analgesia (PCA). In one case, change in route of opioid administration was within the first 6 hours and was used to help with management of ACS rather than for uncontrolled pain. For the five patients whose standard treatment was parenteral opioids, efficacy responses were similar to those whose standard treatment was oral opioids (Supplemental Table 1).

**Table 5.** Efficacy end points.

End point	Sub-MTD SLF 9 episodes	MTD SLF 22 episodes	p-value
Reduction in VAS score from baseline to 6 hours, median (IQR)	2.4 (0.4–5.6)	2.8 (0.0–4.1)	NS
Reduction >1.3 cm in VAS at 6 hours, number (%)	5 (56)	15 (68)	NS
Reduction >2.6 cm in VAS at 6 hours, number (%)	4 (44)	14 (59)	NS
VAS score <5 by 6 hours, number (%)	5 (56)	15 (68)	NS
Discharged from ED/HDU by 6 hours, number (%)	3 (33)	9 (40)	NS
Opioid used during first 6 hours, average oral morphine equivalent in mg/kg (range)	1.1 (0.6–2.0)	1.1 (0.4–1.6)	NS
Time to first dose of analgesia, average minutes (range)	46 (21–76)	53 (27–93)	NS
Duration of hospital stay, days (range)	6.8 (0–28)	2.4 (0–10)	NS
Opioid used during first 24 hours, average oral morphine equivalent in mg/kg (range)	2.1 (0.6–5.2)	2.2 (0.4–7.8)	NS
Opioid used during episode, average oral morphine equivalent in mg/kg (range)	10.8 (0.6–32.6)	13.7 (0.4–189.1)	NS
Conversion to injected opioid, number (%)	2 (22)	4 (10)	NS
Readmission, number (%)	5 (56)	8 (36)	NS

MDT SLF: maximal tolerated dose sublingual fentanyl; IQR: interquartile range; ED: emergency department; HDU: haematology day unit; NS: not significant.

Readmission included ED attendances and hospital admissions. There were a total of 13 readmissions (42% of episodes) of which 6 were within 7 days (19%) and 7 within 14 days (23%). Duration of hospital stay and readmission rate were lower in patients on MTD SLF compared with sub-MTD SLF, but these differences were non-significant (Table 5).

### Safety

**Figure 3.**
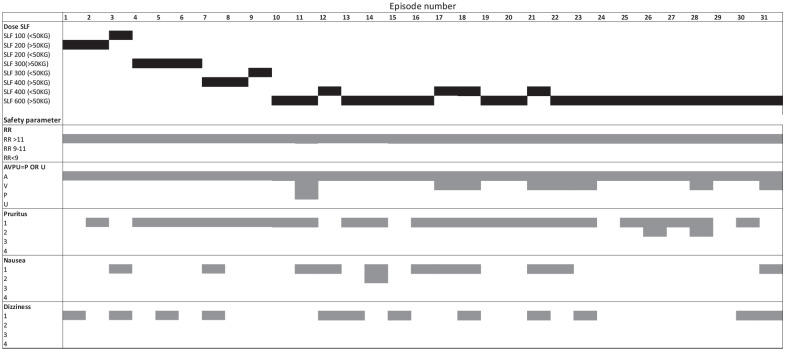
Sublingual fentanyl dosing and opioid adverse events (during the first 6 hours of monitoring) are shown diagrammatically for each treatment episode. MTD episodes are episodes 10–31. In the top section, the black bar represents the dose of SFL given for the treatment episode. In the second and third sections, the most severe degree of respiratory depression and sedation, and in the lower sections, most severe grades of pruritis, nausea and dizziness for each episode are shown as grey bars. SLF: sublingual fentanyl (dose in micrograms); RR: respiratory rate; A: alert; V: responds to verbal commands; P: responds to painful stimuli; U: unresponsive.

Opioid adverse effects observed during the first 6 hours of treatment are illustrated in Figure 3. These were generally mild or moderate. There were no cases of RR below 11 per minute, which would be contributed to a higher risk score in the NEWS system. At MTD, there was one case of severe sedation (P) in a patient who presented to ED with evidence of ACS prior to dosing. MTD was also associated with more cases of mild sedation (V) on the AVPU scale. The majority of participants reported pruritis grade 1 at doses of 300/200 mcg and above. Grade 1 nausea was also more common at MTD SLF. There was no difference in symptoms of dizziness and one case of self-limiting urinary retention at the highest dose.

Serious adverse events (SAEs) were observed in seven episodes in six patients. Four SAEs were due to prolonged hospitalisation, and in these cases, there were no additional complications of SCD. There were three cases of ACS in two patients. The first presented with symptoms of ACS prior to receiving study medication. This patient underwent emergency exchange transfusion and was switched from study analgesia protocol to intravenous fentanyl PCA at 3 hours, making a rapid recovery. A second patient presented with uncomplicated pain episode and was treated with the study protocol and made a rapid recovery. This patient represented on two further occasions over the next 2 weeks, on the second occasion had a rapid deterioration in respiratory function and died. During the second and third admissions, the patient was treated with a standard institution analgesia protocol. The enquiry into this tragic fatal event identified delay in recognising ACS and instituting emergency exchange transfusion. Since the event occurred 10 days after administration of a single dose of SLF, it was not considered related to study medication or procedures.

### Patients dosed on more than one occasion

The protocol allowed up to two treatment episodes per patient in Part 1 and a total of three treatment episodes per patient in total. During the study, five patients were treated more than once (three patients treated three times and two patients treated twice). All 13 of these treatment episodes were at MTD SLF. There was marked variability in efficacy when episodes within an individual patient were compared. None of the patients treated for repeated episodes were observed to have significant opioid adverse events (Supplemental Table 1). One patient experienced ACS, related to two of the three treatment episodes.

### Participant satisfaction

Feedback on the standard protocol questionnaire was obtained 28 days after discharge for 25 episodes in 19 patients. Feedback was not available for six episodes, because patient was uncontactable (5) or patient deceased (1). In 23 of 25 (92%) episodes surveyed, the patient stated they would like to receive the protocol again. Additional written feedback was received which was highly supportive of the protocol (Supplementary Appendix 2).

## Discussion

We have shown that a single dose of SLF at 400 mcg (<50 kg) or 600 mcg (>60 kg) when combined with a pre-scheduled dosing of oral oxycodone is safe for adolescents and adults and can be used for initial management of APC in the acute care setting.

There is an evidence gap in management of APC which has been acknowledged by the NHLBI in the United States^7^ and by the NICE in the United Kingdom.^4^ Our protocol addresses some of the areas of uncertainty about optimal care, including alternative routes of opioid administration, and treatment in the adolescent and young adult age group.

With regard to safety, patients were carefully evaluated over the first 6 hours of observation. There was no evidence of significant over-sedation or respiratory depression, the primary concerns with use of strong opioids. Reporting of other adverse effects was as expected, with mild pruritis, nausea and dizziness frequently reported, mostly at mild severity. ACS was reported in three episodes (9.6%), but none were considered causally related to the analgesia protocol. Rates of ACS in previous studies have been variable, ranging from 3% to 57%,^9,23–26^ and our results do not suggest a higher rate of ACS with oral opiates. We suggest that the risk of ACS associated with oral opioids, as reported in a study of oral versus intravenous morphine, is probably overstated.^9^

Although not formally designed to evaluate efficacy, the data suggest similar efficacy to other analgesia protocols used in SCD. Changes in the VAS pain score over the first 6 hours of treatment or until decision on disposition in ED have previously been evaluated,^27–29^ leading to the suggestion that a change in pain intensity between 1.3 and 2.6 cm on a 10-cm VAS scale would be considered clinically significant.^30^ For episodes treated with MTD SLF in this study, the data suggest a clinically significant reduction in pain, with median reduction of 2.8 cm and 59% of episodes demonstrating at least a 2.6-cm reduction in VAS at the 6-hour time point. In comparable trials of analgesia, the mean reduction was in the range of 2–4 cm.^27–29^ We observed that most of the efficacy measures evaluated showed greater efficacy of MTD SFL over sub-MTD SLF, and this supports a policy of administering the highest safe dose of rapid-acting opioid analgesia.

In our experience, some patients continue to experience pain in the first 1–2 weeks after discharge, and in some cases require readmission to hospital. ED re-attendance and hospital readmission rates were high, but similar to recent studies of patients treated with intravenous opioids in the United States of 35–50%.^27,31^ It is not yet clear how the readmission rate relates to efficacy and acceptability of the study analgesia and we plan to investigate this end point in a randomised controlled study. The evaluation at 28 days was intended to enable full recovery from the episode and withdrawal of analgesic medications which could interfere with judgement of satisfaction. In general, patients had a good recollection of their experience during treatment after the 28-day interval. Participant feedback was generally very positive, and a high proportion of those surveyed wished to be treated again with the same protocol. In an ancillary study where a group of our service users participated in developing a questionnaire to evaluate patient satisfaction with pain management in hospital, we found that satisfaction was not primarily determined by the analgesia protocol, but by the quality of communication, and attitudes of staff in ED and medical wards.^20^ The benefit of one-to-one nursing care given by the trial nurse during the first 6 hours of the protocol was particularly notable in this study. This suggests that continued communication, reassurance and support sustained over the first 6 hours of management in ED and, if admitted, on transfer to the hospital ward were highly valued and contributed to the overall patient-reported outcome. Resources should be directed to this aspect of care.

The protocol was developed following our experience with use of combined IND and short-acting oral morphine in children. We demonstrated that the first dose of IND could usually be delivered by ED staff well within the recommended time limit.^14,19^ In this study, we did not attempt to address timeliness of this first dose, recognising that in the trial setting, delays occur in assessing eligibility, obtaining consent and administering an investigational medicinal product. In order to confirm a satisfactory performance with regard to time to first analgesia, the protocol would need to be formally implemented with training of acute care staff, and further evaluation undertaken by auditing outcomes during routine care.

In a previous study with oral opioid protocols, we found that dosing needed to be proactive in the first 6 hours and scheduled doses given even if pain is at the mild end of the scale (VAS score 1–3), to avoid relapse.^14^ In this study, some patients became drowsy (AVPU score of ‘V’) with repeated doses of oral oxycodone, and some scheduled doses were omitted on the decision of the trial physician. We would therefore suggest a revision to the protocol with scheduled dose of oxycodone omitted if VAS pain score is <3.

Patients who used strong opioids on a daily basis were excluded from the study, and only 38% of patients treated in this study used strong opioids episodically for home management of acute pain, compared to 75% in some adult studies in the United States.^11,32^ The protocol is unlikely to be successful for patients who are already heavily exposed to opioids, and may not be acceptable to some patients who are already established on intravenous protocols for pain management in hospital. We suggest that a comparative trial would be of most value in adolescent and young adult populations who are not frequent attenders to hospital and are not yet heavily exposed to opioids.

In conclusion, these results provide evidence that the study protocol is safe, acceptable and potentially effective for initial pain management of APC in adolescents and adults. Use of SLF on arrival in ED, combined with pre-scheduled oral opioid, could reduce time to first analgesia as well as preventing short- and long-term complications associated with repeated use of intravenous opioids. We suggest that the protocol should be further evaluated in different health care settings, including supervised treatment at home, as well as being formally compared to protocols based on injected opioids.

## Supplemental Material

sj-pdf-1-bjp-10.1177_20494637211033814 – Supplemental material for A non-injected opioid analgesia protocol for acute pain crisis in adolescents and adults with sickle cell disease Click here for additional data file. Supplemental material, sj-pdf-1-bjp-10.1177_20494637211033814 for A non-injected opioid analgesia protocol for acute pain crisis in adolescents and adults with sickle cell disease by Paul Telfer, Jonathan Bestwick, James Elander, Arlene Osias, Nosheen Khalid, Imogen Skene, Ruben Nzouakou, Joanne Challands, Filipa Barroso and Banu Kaya in British Journal of Pain
