# Supplementary material for: A non-injected opioid analgesia protocol for acute pain crisis in adolescents and adults with sickle cell disease
Source: Br J Pain. 2021 Aug 2;16(2):179–90. doi: 10.1177/20494637211033814 (PMC8998522; doi:10.1177/20494637211033814)
Supplement: sj-pdf-1-bjp-10.1177_20494637211033814 – Supplemental material for A non-injected opioid analgesia protocol for acute pain crisis in adolescents and adults with sickle cell disease [file sj-pdf-1-bjp-10.1177_20494637211033814.pdf]

Supplemental material

Appendix 1:

Protocol amendments

The following protocol amendments were made during the trial:

(i) To include other opiate adverse effects (itching, nausea, dizziness, urinary retention and constipation) graded on a 0-4 scale in the Trial Safety Monitoring Committee (TSMC) decision making on dose change.

(ii) To allow the same dose to be used as an alternative option to an increase or reduction in determination of maximum tolerated dose.

(iii) Dose of oxycodone at t=6 hours brought forward to t=5 hours after consultation with TSMC, to improve efficacy of analgesia and regularise the interval between oxycodone doses to 2 hourly.

(iv) In view of concerns about other opioid adverse effects at iteration +3, the TSMC decided that the dose for the final iteration (+4) might be too high, especially for young patients and opioid naïve patients, and was therefore not used in the study .

(v) The number of adjudicated episodes was reduced to 21 in view of the reduced number of iterations and in order to ensure timely completion of the trial.

(vi) Initially, the age range was 12-60 years, but a protocol amendment was implemented to increase the entry age to 14 years in the early stages of the study because of concerns about including younger opioid-naïve children in a fixed dosing schedule.

Appendix 2: Written feedback from patients

'Being on the SCAPE trial and having someone monitor me throughout the trial was very useful and successful. Overall, I noticed that the scape trial worked really well and was fast with the pain, it helped ease my crisis and was a complete success with how it went, I am glad that I agreed to be on the SCAPE trial as it benefited me and I feel that it could help others with sickle cell as well'

'I did find (Trial nurse) being there helpful as she was always on hand if I needed anything, my pain was managed well too as she was always checking on me, making sure I was ok, so I did find it helpful. She was very attentive and had good communication and because of that I think my pain was managed well.'

'Being on the SCAPE trial and having a delegated nurse, in my opinion, was effective as once I got to A&E I was called through and did not wait long for the nurse and the doctor to attend to me
(I did call the SCAPE team in advance to let them know I was on my way). It allows patients like myself to be aware of what's going on, and what to expect with each medication and the side effects. It also allows patients to ask questions or discuss any concerns they may have with their nurse. As well as providing any feedbacks. I did not have any issues or concerns as I was always informed on what was happening and why, and what to expect next. Communication was ongoing with myself and the nurse. I think all Sickle cells patients will benefit from this greatly.'

'Recently I participated in the SCAPE trial which required a Research Nurse to be responsible and available for my treatment all throughout the work day. The impact of this was amazing as it meant I was able to receive my treatment on time every time, which pushed me to full recovery much more quickly. I can state now through such experience that all of us with similar conditions would benefit greatly from this sort of treatment, from having a specific Pain Nurse to be responsible for this one essential element of care.'

Supplemental Table 1. Efficacy outcomes and adverse events.

A: First event for individual patient

Patient number	1	2	3	4	5	6	7	8	9	10	11	12	13	14	15	16	17	18	19	20	21	22	23
Episode number	1	2	3	4	5	6	7	8	9	10	11	12	13	14	15	17	18	19	20	23	27	29	31
**Efficacy**																				1	1		1
Reduction in VAS score from baseline to 6 hours	2.4	1.1	0	5.2	3.9	0.8	-1.1	5.8	5.5	4.6	-0.8	-0.8	5.4	-0.1	5.2	0	-0.2	1.9	4.5	1.3	3.2	3.7	3.6
VAS<5 BY 6 HRS	0	0	0	1	1	0	0	1	1	1	0	0	1	0	1	0	0	0	1	0	1	1	1
Discharged ED by 6 hrs	0	0	0	0	1	0	0	1	1	0	0	0	1	0	1	0	0	0	1	0	1	1	1
Duration of hospital stay (days)	1	15	1	3	0	13	28	0	0	1	4	10	0	3	0	4	3	1	0	2	0	0	0
Opioid used during first 6 hours (morphine eq- mg/kg)	1.23	1.38	0.61	0.70	0.98	0.87	1.22	1.05	0.88	0.79	1.21	1.49	1.51	1.25	0.38	0.36	1.26	0.71	1.10	0.98	0.77	1.43	0.64
Opioid used during first 24 hours (mprphine eg-mg/kg)	3.08	2.77	0.61	1.80	0.98	2.67	4.36	1.05	0.88	1.84	1.47	7.84	1.51	3.33	0.38	0.98	3.77	0.71	1.10	1.63	0.77	1.43	0.64
Opioid used during entire episode (morphine eg- mg/kg)	3.08	23.88	0.61	6.48	0.98	32.57	26.66	1.05	0.88	1.84	7.90	189.14	1.51	7.08	0.38	3.39	9.12	0.71	1.10	1.63	0.77	1.43	0.64
Conversion to injected opioid	0	0	0	1	0	1	0	0	0	0	1	1	0	0	0	0	1	0	0	0	0	0	0
Readmission	1	1	0	1	1	1	0	0	1	0	0	0	0	0	0	0	0	0	1	0	0	1	0
**Adverse effects**																							
Itching	0	1	0	1	1	1	1	1	1	1	0	0	1	1	0	1	0	1	0	1	1	0	0
Nausea	0	0	1	0	0	0	1	0	0	0	0	1	0	2	0	1	0	0	0	0	0	0	1
Dizziness	1	0	1	0	1	0	1	0	0	0	0	0	1	1	0	0	0	0	0	1	0	0	1

Standard care for patients 4,6,13,18,19 was with injected opioids

B: Patients treated for multiple events

Patient number	11			15		17			19			22	
Episode number	11	26	28	15	16	18	21	22	20	24	25	29	30
**Efficacy**													
Reduction in VAS score from baseline to 6 hours	-0.8	3.2	2.3	5.2	5.3	1.9	2.7	0.2	4.5	-0.9	4	3.7	3
VAS<5 BY 6 HRS	0	1	0	1	1	0	1	0	1	0	1	1	1
Discharged ED by 6 hrs	0	1	0	1	0	0	1	0	1	0	0	1	1
Duration of hospital stay (days)	4	0	5	0	5	3	0	8	0	3	4	0	0
Opioid used during first 6 hours (morphine eq- mg/kg)	1.21	1.18	1.55	0.38	1.15	1.26	0.96	0.96	1.10	1.11	1.10	1.43	1.43
Opioid used during first 24 hours (mprphine eg-mg/kg)	1.47	1.18	3.10	0.38	3.08	3.77	0.96	2.24	1.10	4.81	3.38	1.43	1.43
Opioid used during entire episode (morphine eg- mg/kg)	7.90	1.18	23.88	0.38	11.41	9.12	0.96	6.40	1.10	12.69	15.82	1.43	1.43
Conversion to injected opioid	1	0	1	0	0	1	0	0	0	0	0	0	0
Readmission	0	1	0	0	0	0	1	0	1	1	1	1	1
**Adverse effects**													
Itching	0	0	0	0	0	0	0	0	0	0	0	0	0
Nausea	0	0	0	0	0	0	0	0	0	0	0	0	0
Dizziness	0	0	0	0	0	0	0	0	0	0	0	0	0
